# Visual search in neurodevelopmental disorders: evidence towards a continuum of impairment

**DOI:** 10.1007/s00787-021-01756-z

**Published:** 2021-03-22

**Authors:** Daniela Canu, Chara Ioannou, Katarina Müller, Berthold Martin, Christian Fleischhaker, Monica Biscaldi, André Beauducel, Nikolaos Smyrnis, Ludger Tebartz van Elst, Christoph Klein

**Affiliations:** 1 Department of Child and Adolescent Psychiatry, Psychotherapy, and Psychosomatics, Medical Center - University of Freiburg, Faculty of Medicine, University of Freiburg, Freiburg, Germany; 2 Psychotherapeutisches Wohnheim für junge Menschen Leppermühle, Buseck, Germany; 3 Institute of Psychology, University of Bonn, Bonn, Germany; 4 2nd Psychiatry Department, National and Kapodistrian University of Athens, Medical School, University General Hospital “ATTIKON”, Athens, Greece; 5 Department of Psychiatry and Psychotherapy, Medical Center - University of Freiburg, Faculty of Medicine, University of Freiburg, Freiburg, Germany; 6 Department of Child and Adolescent Psychiatry, Medical Faculty, University of Cologne, Cologne, Germany

**Keywords:** Schizophrenia, Attention-Deficit/Hyperactivity Disorder (ADHD), Autism Spectrum Disorder (ASD), Serial visual search, Eye movement

## Abstract

Disorders with neurodevelopmental aetiology such as Attention-Deficit/Hyperactivity Disorder (ADHD), Autism Spectrum Disorder (ASD) and Schizophrenia share commonalities at many levels of investigation despite phenotypic differences. Evidence of genetic overlap has led to the concept of a continuum of neurodevelopmental impairment along which these disorders can be positioned in aetiological, pathophysiological and developmental features. This concept requires their simultaneous comparison at different levels, which has not been accomplished so far. Given that cognitive impairments are core to the pathophysiology of these disorders, we provide for the first time differentiated head-to-head comparisons in a complex cognitive function, visual search, decomposing the task with eye movement-based process analyses. *N* = 103 late-adolescents with schizophrenia, ADHD, ASD and healthy controls took a serial visual search task, while their eye movements were recorded. Patients with schizophrenia presented the greatest level of impairment across different phases of search, followed by patients with ADHD, who shared with patients with schizophrenia elevated intra-subject variability in the pre-search stage. ASD was the least impaired group, but similar to schizophrenia in post-search processes and to schizophrenia and ADHD in pre-search processes and fixation duration while scanning the items. Importantly, the profiles of deviancy from controls were highly correlated between all three clinical groups, in line with the continuum idea. Findings suggest the existence of one common neurodevelopmental continuum of performance for the three disorders, while quantitative differences appear in the level of impairment. Given the relevance of cognitive impairments in these three disorders, we argue in favour of overlapping pathophysiological mechanisms.

## Introduction

With the advent of the DSM-5 [1], renewed conceptualisations of mental disorders lead to the categories of “Neurodevelopmental Disorders” (NDD) and “Schizophrenia Spectrum and Other Psychotic Disorders” (SSD). The term ‘neurodevelopment’ extends to disabilities with strong genetic influences, multifactorial aetiologies, onset in childhood and steady clinical course, in spite of maturational changes [2].

Despite its neurodevelopmental origin [3], schizophrenia has so far been considered as aetiologically and nosologically distinct from other NDD.

However, genomic studies identified rare risk alleles common among NDD, including Autism Spectrum Disorder (ASD), Attention-Deficit/Hyperactivity Disorder (ADHD) and schizophrenia, leading to a model in which NDD are represented in a neurodevelopmental and aetiological continuum, expressing the severity, timing, pattern of abnormal brain development and resulting in functional abnormalities [4].

Further commonalities include shared environmental risk factors, higher frequency in males, frequent comorbidities and overlapping mechanisms at multiple levels. Phenotypically, impairments in various executive functions, in rapid information processing and sustained attention and difficulties in motor coordination are common across the three disorders [5]. Moreover, while adults with schizophrenia share with children with ASD deficits in planning, cognitive flexibility, social cognition, social competence and sensory-motor problems, they share with children with ADHD deficits in inhibition, which seem to be less pervasive in children with ASD. Finally, ADHD and ASD share deficits in attention, working-memory, processing speed, emotion regulation, but not verbal and non-verbal communication skills, which seem to be preserved in ADHD [5–7]. Neurobiologically, reduced brain volume has been reported at the level of default mode network (DMN) in ASD and ADHD, frontoparietal and limbic networks in schizophrenia and ASD, ventral attention network in ADHD and schizophrenia [8]. In ASD, as opposed to ADHD, larger total brain and white matter volumes in most cortical and some subcortical brain regions were found, while schizophrenia and ADHD show different developmental trajectories, with parallel growth curves for ADHD and progressive decline in region-specific grey matter loss for schizophrenia [5].

Among the phenotypic commonalities cognitive impairments play a prominent role as they are in all these disorders crucial for functional outcome and may pinpoint part of the underlying pathophysiology.

Visual search (VS) is a frequently studied paradigm in cognitive research and includes the dichotomy between parallel and serial search. When the target is easily distinguishable from non-target items (“distractors”), items are searched in parallel and search efficiency is independent of the number of distractors. Conversely, when the target is hardly distinguishable from distractors, attention is narrowed down, items are searched in serial fashion and search efficiency decreases linearly with the increase in the number of distractors.

 Participants with schizophrenia, ADHD or ASD show normal *parallel* search [9]. By contrast, less efficient *serial* search characterises patients with schizophrenia [10, 11] or ADHD [9], especially with larger search displays. The same deficit was found in unaffected schizophrenia [12] and ADHD [13] first-degree relatives, while in some cases their performance was intermediate between patients and controls [14]. Individuals with autism [15] and their first-degree relatives [16], conversely, show normal or superior-to-normal performance, which may indicate enhanced “local” perceptual discrimination as the flip side of impaired—or less favoured—“global” processing [17].

A methodological commonality of the studies above is the exclusive use of manual mean reaction time (RT) and search rate, collapsing a complex cognitive behaviour into a single measure. In fact, multiple cognitive capacities are involved during serial search, including short-term memory, focus and shift of attention. Arguably, deficits in any of these sub-processes could lead to VS impairment, requiring their differentiation in *process analyses* of VS. Such analyses can be accomplished by eye movement recordings, providing a highly differentiated picture of VS performance, as will be shown here.

VS is located within a research framework, where the measurement of eye movement during standard ocular-motor tasks and visual exploration tasks has contributed to the understanding of the complex underlying neuropathophysiology of NDD. Literature on basic saccade paradigms reported delayed latency, frequent inaccuracy and inhibition errors in the execution of volitional saccades across schizophrenia, ADHD and ASD [18]. A less frequently included measure is intra-subject variability (ISV), as the within-subject moment-to-moment fluctuations in task performance. Robust findings of increased ISV on saccade tasks were reported in schizophrenia [19] and ADHD [20]. Similar findings in ASD are difficult to interpret due to high comorbidities with ADHD that remained undiagnosed before the DSM-5 [21]. Studies on visual exploration in patients with schizophrenia showed restricted visual scan paths by means of reduced saccade amplitude and fixations and reduced exploration of salient social features and abstract non-social features, suggesting a general visual scanning and exploration impairment, independent of the semantic content of an image [22]. Similar findings were reported in children and adolescents with ASD [23, 24] and a group of high-risk for ASD infants [25]. Findings from the literature on ADHD are more conflicting, as some reported more restricted patterns of visual exploration than healthy controls when looking at social and non-social stimuli [26], while some others did not [27]. Under the visual search paradigm, the increase in task complexity of the serial type of search offers an ideal scenario to study potential deficits in cognitive requirements in a trade-off with the understanding of potential atypicalities in the visual-motor exploration of the scene.

Based on the above, the present study investigated VS performance simultaneously in schizophrenia, ADHD and ASD, pursuing the following goals: (i) to determine the differences between clinical groups and of each clinical group with TD; (ii) to explore similarities between clinical groups in their deviances from controls.

The study provides the following hypotheses: (a) patients with schizophrenia will show delayed responses and generalised increased ISV; (b) patients with ADHD will show slower manual RT alongside generalised increased ISV; (c) patients with ASD will outperform all other groups in processing speed, including less fixations and will show increased ISV; (d) patients with schizophrenia will present the overall most impaired performance, ASD the least; (e) profiles of deviancy from controls will be positively correlated between all clinical groups.

The present study is the first “head-to-head” comparison of schizophrenia, ADHD and ASD in core aspects of cognitive proficiency providing process analyses of cognitive performance through eye movement recordings.

## Methods

### Participants

Participants were 20 patients with schizophrenia (SCZ, 15–22 years), 28 with ADHD (18–23 years), 26 with ASD and 29 TD (17–23 years) (Table 1). None of them had epilepsy or neurological diseases, all had normal or corrected-to-normal vision. SCZ were recruited from the rehabilitation centre Psychotherapeutisches Wohnheim für junge Menschen Leppermühle and had a diagnosis of schizophrenia, schizophreniform or schizoaffective disorder, for which they were receiving antipsychotics (Table 2). One patient with schizophrenia had also received a diagnosis of ADHD, for which he was, however, not taking psychostimulant medication. Given that repeating the analysis excluding such participant did not produce any difference in the findings in any dependent variable, data from this participant were included in the final analysis. Patients with ASD or ADHD were recruited from the out-patient populations of the Department of Child and Adolescent Psychiatry, Psychotherapy, and Psychosomatics, Medical Centre, University of Freiburg, while candidate participants with comorbid ADHD and ASD were excluded. ASD diagnosis was determined using the Autism Diagnostic Observation Schedule (ADOS; [28]) and the Autism Diagnostic Interview-Revised (ADI-R; [29]). ADHD diagnosis was based on interviews, clinical observations and the Conner’s Rating Scales [30]. In addition, the Social Responsiveness Scale (SRS; [31]) and the Conners’ Rating Scales assessed the specificity and the severity of ASD and ADHD symptoms, respectively, in ASD and ADHD and their absence in TD. Participants taking methylphenidate psychostimulants were medication-free for at least 24 h prior to testing, a period considered sufficient for the release of medication effect [32]. Controls were recruited through the department’s database by posting announcements and by word of mouth. The absence of a family history of psychiatric disorders and present or past own psychiatric disorders was verified prior to participation. The CFT 20-R [33] for ASD/ADHD/TD and the Wechsler Intelligence Test (WISC-IV, WAIS-IV; [34]) for SCZ were used to measure IQ. Additionally, a 9-item version of the Raven Standard Progressive Matrices (RSPM; [35]), correlating with the original version by *r* = 0.98 [36], was administered. A dominant eye test determined the ocular preference, handedness was assessed with the Edinburgh Handedness Inventory [37]. The study was given ethical approval by the University of Freiburg Ethics Committee. Adult participants provided written informed consent. For minors both parents’ and their written informed consent was obtained.

**Table 1:** Group’s characteristics

Variable	SCZ	ADHD	ASD	TD	*F*_3,99_	*p*^a^
*N*	20	28	26	29		
Mean age ± standard deviation (SD)	19.8 ± 1.7	19.9 ± 1.4	19.7 ± 1.9	19.8 ± 1.6	0.094	0.979
Gender (% female)	29	46	4	59	7.834	**0.001**
RSPM (% correct)	47.2 ± 27.2	58.3 ± 25.8	72.2 ± 19.7	69.0 ± 16.4	5.920	**0.001**
Current medications (%)	100	28.6	0	–	–	–

^a^Significant *p* values are shown in bold

**Table 2:** Medication type and dose in SCZ

*N*	Medication type	Dose (mg)
11	Clozapine	150–400
5	Aripiprazole	2.5–20
3	Olanzapine	5–10
3	Quetiapine	300–400
6	Pipamperone	20–60
3	Risperidone	0.5–4.5
4	Venlafaxine	150–225
3	Fluoxetine	20
3	Escitalopram	20

*N* = number of patients

### Procedure

Participants were tested individually inside a lit cabin using a chinrest. They faced a flat, 24 in. LCD monitor with 1920 × 1080 pixels resolution and 60 Hz refresh rate, located 90 cm in front of them, connected to a PC running Experiment Builder (SR Research Ltd., version 2.1.140). The illumination level was measured by a digital Peaktech 5035 light meter with range 0–2000 lx (Ahrensburg, Germany), maintained between 70 and 80 lx across participants. Eye movements were recorded binocularly at 1000 Hz sampling rate and 0.01° spatial resolution, using the EyeLink 1000 Plus (SR Research, Mississauga, ON, Canada). A thirteen-point manual calibration was used. Gaze positions were calibrated if gaze accuracy was within 1°. Before each trial participants performed a drift correction, ascertaining that gaze accuracy was within 0.5°. The VS task was presented during a 150-min session, including three 10-min breaks, as part of a battery of several other eye movement tasks. The order in which tasks were presented was counter-balanced across participants within each group through a balanced Latin Square, thus obtaining a constant task order across the four groups and preventing task order from being a confounding variable. Participants were rewarded for their participation with vouchers.

### Visual search

Search displays consisted of 16 items, corresponding to 0.90° × 0.90° black outlined squares (line width 0.16°) with a 0.17° gap on one of the sides. Distractors had gaps on either left or right side, targets on either top or down side. Items were placed in a 4 × 4 grid (18° × 18°), target in eight out of 16 locations, four with shortest and four with longest distance from the centre of the grid, resulting in two eccentricities and thus different retinal visual acuities [38]. Targets were presented in the same location in four non-consecutive trials with pseudo-randomized trial order. The task consisted of 32 trials plus four practice trials (Table 3).

**Table 3:** Illustration of task and search phases, description of task, parameters

Illustration	Task/task phases description and parameters
	*Task*—participants had to locate the single item having either an upward or downward facing gap, as rapidly as possible *Parameters*—percentage of correct responses, as the percent of the number of correct responses out of the total number of trials; percent of time that the eye was missing per trial; manual reaction time (RT), as the time between the onset of the search grid and the Ctrl-Left button press; total number of fixations on the grid; fixation duration and saccade amplitude
	*Task phase*—trial started with a central cross, which disappeared when gaze was detected within an area of 1.5° for at least 1000 ms. The search array followed and remained on the grid for 8000 ms, or until button press *Parameters*—none
	*Task phase*—initiation of search, as the time between the offset of the first fixation and the onset of the first saccade on the grid *Parameters*—latency of the first saccade on the search grid
	*Task phase*—search, as the time between onset of the first saccade on the grid and onset of the last fixation to the target. Within search, scanning time, until onset of the first fixation on target *Parameters*—total search duration; scanning time: duration; scanning pattern (sum distance covered by all saccades from grid onset to the first fixation on target); fixation duration (duration for all fixations); frequency of fixations, out of the total number of fixated distractors; saccade amplitude (amplitude for all saccades)
	*Task phase*—post-search. Press Ctrl-Left as quickly as possible as soon as target has been found, then press button 1–4 according to the column in which the target is *Parameters*—post-search duration

Mean and SD were calculated for all parameters, except frequency of fixations

### Analyses of ocular-motor data

Analyses were performed on one eye’s data, depending on ocular preference and the eye trace quality. Despite binocular recording, we opted for monocular analysis because of the discrepancy in the eye trace quality between the two eyes in some participants. The decision on which eye’s data to use was based on the offline evaluation of first calibration, validation, drift corrections and additional possible calibrations alongside reported ocular preference. Saccades were detected through a velocity-based method (EyeLink algorithm). “Fixation” was defined as any period that was neither blink nor saccade. A blink was inferred any time the tracking of the eye was lost, resulting from occlusion of the eye by the eyelid. Only saccades with peak velocities above 25°/s and amplitudes greater than 1° were analysed. Responses after 8000 ms were counted as errors. Anticipatory responses were initiated prior to, or within 80 ms from search grid appearance.

### Statistical analyses

**Fig. 1:**
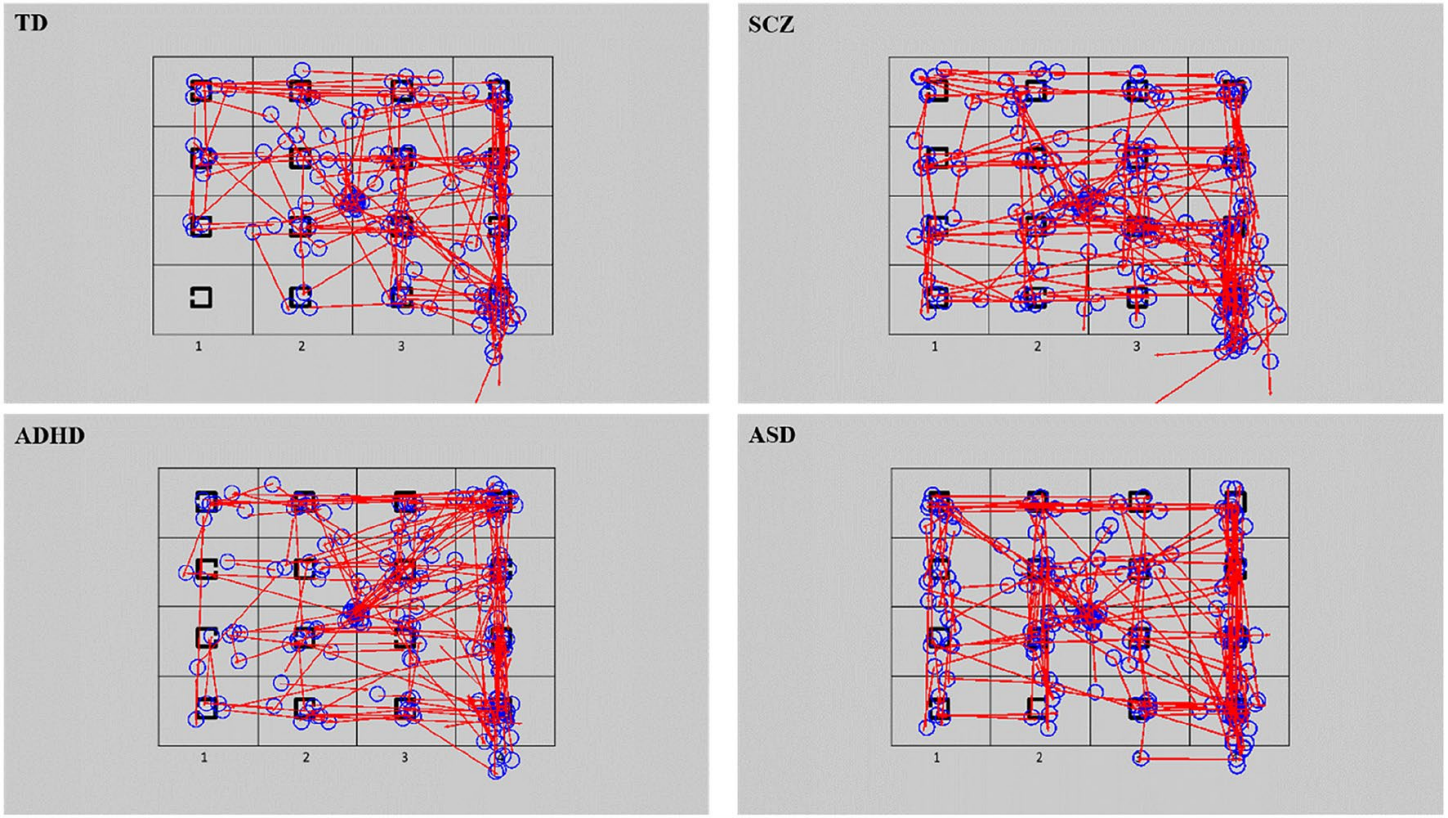
Scan paths for the four groups, based on one trial, aggregated for 20 participants per group, randomly selected. Blue circles are the fixations, whose diameter is based on fixation duration, red lines are the saccades

Dependent variables are described in Table 3. Scan paths are presented in Fig. 1. Group differences were analysed by means of ANOVA with GROUP as between-subject factor with four levels—TD, SCZ, ADHD, ASD. Planned contrasts using *t* tests allowed for comparisons of each clinical group with TD, while non-predicted pair-wise comparisons were computed using post-hoc Tukey’s test. Repeated measures ANOVA allowed to explore interactions between GROUP and TARGET ECCENTRICITY (near, far). Results remained essentially the same when age and RSPM scores were controlled for statistically. Therefore, only results without covariates are reported. Gender was also not included as a covariate as control analyses comparing male and female participants revealed no significant differences. To control for the potential effect of antipsychotic medications in the SCZ group, each dependent variable was correlated with the antipsychotic dose, using Pearson’s *r*. To do so, the medication dose was converted to chlorpromazine equivalents (CPE) per day [39, 40]. For those patients on multiple antipsychotics, the CPE converted medication doses were summed up within participant.

Importantly, we ran vector correlations using Pearson’s *r* to precise the degree of similarity of profiles of deviancy from controls between pairs of clinical groups. Each vector reflects the between-group difference (*t* test scores) of each clinical group from TD across the set of dependent measures, thus obtaining three vectors (SCZ-vs-TD; ADHD-vs-TD; ASD-vs-TD). For all measures of SD, these *t* tests were based in addition on the residuals after controlling for the corresponding means, thus addressing the known problem of correlations between central tendency and dispersion. Finally, and to investigate differences in the degree of impairment between clinical groups, we defined vector levels. To do so, the average of each of the three vectors was used to quantify the (average) level of impairment of each clinical group by calculating the paired-sample *t* test between pairs of vectors.

For the ANOVAs statistical analyses, a significance level of *α* = 0.05 was adopted, Cohen’s *d* quantified effect sizes. Analyses were performed with SPSS, Version 24 (SPSS Institute Inc., Cary, NC, USA).

## Results

Descriptive statistics are reported in Table 4.

**Table 4:** Descriptive statistics for all dependent variables by Group

Variable	SCZ	ADHD	ASD	TD
	Mean (SD)	Mean (SD)	Mean (SD)	Mean (SD)
Mean % correct responses	95.7 (8.0)	99.4 (1.2)	99.6 (1.0)	99.5 (1.2)
Mean % missing data	15.1 (14.7)	8.1 (10.5)	7.0 (12.5)	2.0 (4.1)
Mean manual RT	2761.8 (723.2)	2344.3 (540.8)	2030.1 (528.2)	2205.4 (386.2)
SD manual RT	1227.2 (288.1)	1131.0 (280.3)	922.2 (294.1)	1067.6 (262.3)
Frequency fixations	11.3 (2.3)	10.5 (2.3)	8.7 (2.3)	10.1 (1.7)
Mean fixation duration	198.8 (22.1)	190.4 (18.4)	211.2 (41.9)	135.9 (60.8)
Mean of SD fixation duration	90.6 (17.1)	75.1 (11.0)	93.4 (29.1)	39.8 (36.3)
Mean initiation of search	304.2 (52.5)	278.8 (31.1)	299.1 (61.3)	263.5 (43.5)
SD initiation of search	74.3 (24.5)	64.0 (22.5)	77.5 (34.3)	47.6 (16.4)
Mean search	1993.7 (581.0)	1722.9 (456.2)	1396.3 (461.7)	1673 (349.3)
SD search	1250.7 (273.4)	1123.7 (290.0)	913.6 (280.7)	1070.3 (266.4)
Mean scanning time	1732.7 (468.6)	1529.1 (405.1)	1229.0 (412.7)	1526.7 (325.9)
SD scanning time	1223.2 (301.7)	1073.8 (277.0)	893.8 (245.6)	1055.2 (259.8)
Mean scanning pattern	31.7 (8.9)	29.9 (7.6)	23.3 (7.0)	29.9 (19.6)
SD scanning pattern	22.3 (4.9)	20.3 (4.8)	17.9 (4.9)	20.8 (5.7)
Frequency distractors	6.2 (1.8)	5.9 (1.4)	4.6 (1.5)	6.2 (1.3)
Mean post-search	496.3 (212.0)	358.8 (119.2)	395.3 (158.5)	298.8 (72.3)
SD post-search	216.8 (101.4)	160.0 (60.1)	193.9 (83.9)	141.6 (64.9)

Manual RTs were slower and search durations longer on trials with far targets, while no other differences were found. Despite accuracy being above 90% across groups, SCZ were more error prone than TD, ADHD or ASD, whereas the other groups did not differ (*t*s < 0.1). While all trials associated with a correct response were classified as valid and thus included in the analyses, the average percent of time in which the eye was missing was rated 2 ± 4% for TD, 15 ± 15% for SCZ, 8 ± 11% for ADHD and 7 ± 16% for ASD, significantly higher in SCZ and ADHD than TD, only marginally higher in ASD than TD, while not discriminating clinical groups.

**Fig. 2:**
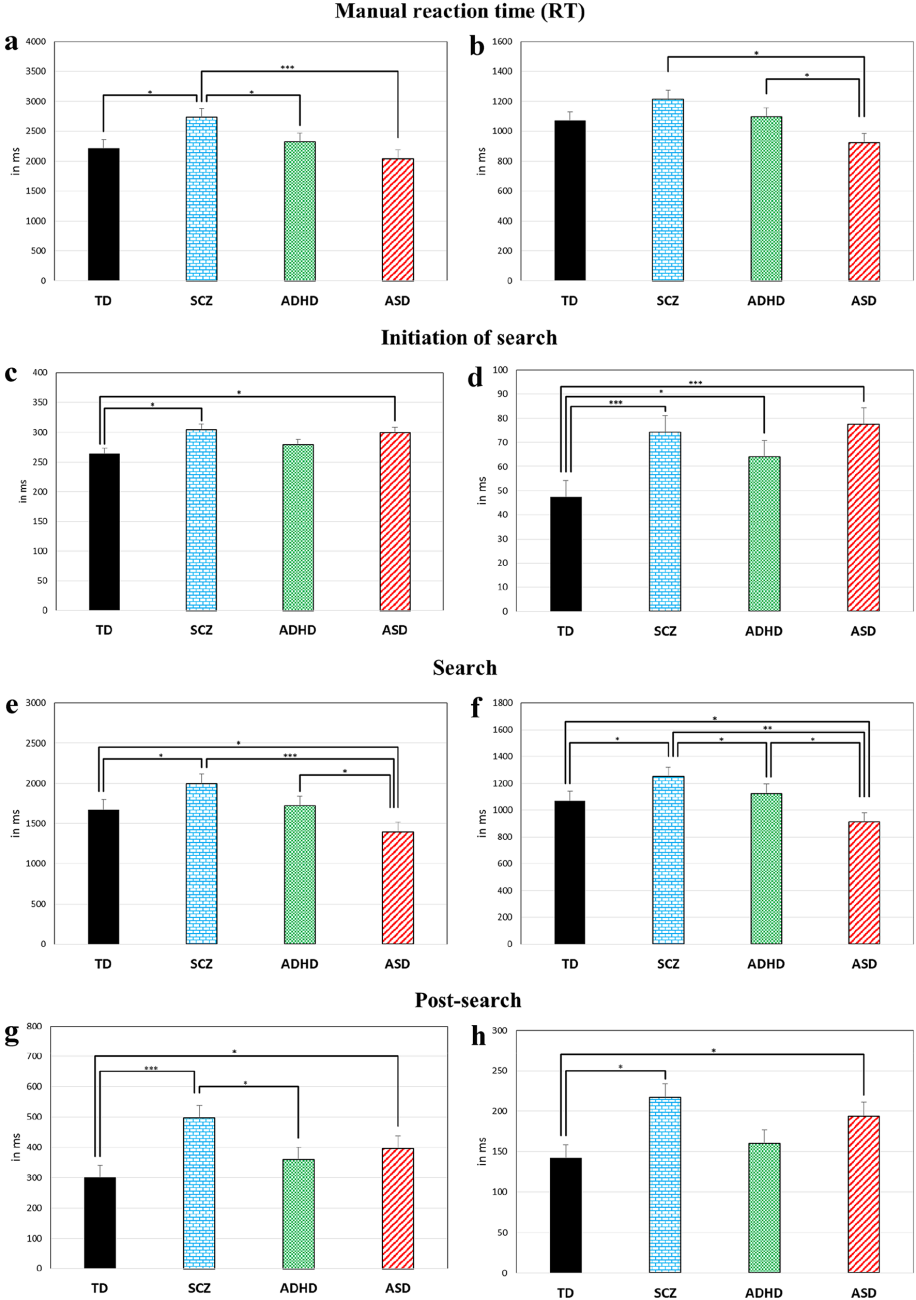
Mean (**a**, **c**, **e**, **g**) and SD (**b**, **d**, **f**, **h**) of manual response times (in ms) and response time for the three search sub-phases. Error bars represent one standard error of the mean. *p ≤ 0.05, **p ≤ 0.001, ***p ≤ 0.0001

The following results are based on trials with correct responses. Table 5 provides between-group ANOVAs for all dependent variables, Fig. 2 provides bar charts for between-group differences in manual RT, initiation of search, search and post-search.

**Table 5:** Between group difference Anova

Dependent variable	F_99_	*p*^a^	*η*_*p*_^2^
Mean % correct responses	5.913	**0.001**	0.155
Mean % missing data	5.858	**0.001**	0.151
Mean manual RT	6.596	**< 0.0001**	0.182
SD manual RT	4.906	**0.003**	0.129
Mean initiation of search	3.952	**0.010**	0.107
SD initiation of search	7.784	**< 0.0001**	0.191
Frequency fixations	6.280	**0.001**	0.160
Mean fixation duration	18.199	**< 0.0001**	0.355
Mean of SD fixation duration	7.161	**< 0.0001**	0.424
Mean scanning pattern	5.848	**0.001**	0.151
SD scanning pattern	3.037	**0.033**	0.084
Frequency distractors	6.273	**0.001**	0.160
Mean search	6.532	**< 0.0001**	0.165
SD search	5.865	**0.001**	0.151
Mean scanning time	6.314	**0.001**	0.161
SD scanning time	5.733	**0.001**	0.148
Mean post-search	7.874	**< 0.0001**	0.193
SD post-search	4.650	**0.004**	0.123

^a^Significant *p* values are shown in bold

*SCZ* were impaired across several facets and measures of VS (Table 6). First, they needed more time than TD in providing a manual response. They were also slower than TD in initiating search, search and post-search, while not being slower in scanning time. Second, while increased ISV characterized SCZ only descriptively for manual RT, significantly higher ISV in SCZ than TD was found during search initiation, search, scanning time and post-search. Third, SCZ showed longer and more variable fixation durations, compared to TD, while they did not differ from TD in the number of fixated distractors and in mean and SD of scanning pattern.

**Table 6:** Planned contrasts between TD and clinical groups

Dependent variable	SCZ			ADHD			ASD		
	*t*_99_	*p*^a^	*d*	*t*_99_	*p*^a^	*d*	*t*_99_	*p*^a^	*d*
Mean % correct responses	3.598	**0.001**	0.733	0.021	0.984	0.016	0.187	0.852	0.159
Mean % missing data	4.173	**< 0.0001**	1.325	2.129	**0.036**	0.764	1.692	0.094	0.54
Mean manual RT	3.234	**0.002**	0.935	0.719	0.474	0.218	1.210	0.229	0.385
SD manual RT	1.821	0.072	0.538	0.380	0.705	0.105	1.980	0.051	0.526
Mean initiation of search	2.934	**0.004**	0.859	1.210	0.229	0.403	2.761	**0.007**	0.676
SD initiation of search	3.671	**< 0.0001**	1.331	2.479	**0.015**	0.838	4.430	** < 0.0001**	1.133
Frequency fixations	/			/			2.365	**0.020**	0.678
Mean search	2.403	**0.018**	0.668	0.405	0.686	0.121	2.242	**0.027**	0.683
SD search	2.232	**0.028**	0.67	0.725	0.470	0.192	2.089	**0.039**	0.574
Mean scanning time	1.772	0.080	0.528	0.022	0.983	0.006	2.756	**0.007**	0.806
SD scanning time	2.143	**0.035**	0.605	0.260	0.795	0.069	2.217	**0.029**	0.638
Mean post-search	4.767	**< 0.0001**	1.354	1.590	0.115	0.612	2.508	**0.014**	0.799
SD post-search	3.358	**0.004**	0.92	0.904	0.368	0.294	2.513	**0.014**	0.702

“/” = no planned contrast specified

^a^Significant *p* values are shown in bold

*ADHD* performance was often in between SCZ and TD (Tables 6, 7, 8). First, ADHD were as fast as TD in manually responding but faster than SCZ. They did not differ from TD in mean duration of initiating search, search, scanning time and post-search while being faster than SCZ during post-search. Second, ADHD were not more variable than either TD or SCZ in manual RT. Conversely, they were more variable than TD, similarly to SCZ, in initiating search. Furthermore, they did not differ from TD and SCZ in ISV of search, scanning time and post-search. Third, they did not differ from either TD or SCZ on average frequency of fixations and of fixated distractors and on mean and SD of scanning pattern, but showed longer and more variable single fixation duration than TD, similarly to SCZ.

**Table 7:** Post-Hoc Tukey’s test between TD and clinical groups

Dependent variable	SCZ			ADHD			ASD		
	*t*_99_	*p*^a^	*d*	*t*_99_	*p*^a^	*d*	*t*_99_	*p*^a^	*d*
Frequency fixations	2.028	0.185	0.638	0.813	0.848	0.228	/		
Mean fixation duration	5.291	**< 0.0001**	1.285	5.030	**< 0.0001**	1.206	6.811	**< 0.0001**	1.428
Mean of SD fixation duration	6.729	**< 0.0001**	1.691	5.133	**< 0.0001**	1.308	7.644	**< 0.0001**	1.621
Mean scanning pattern	0.804	0.852	0.224	0.068	1.000	0.019	3.223	**0.009**	0.933
SD scanning pattern	0.982	0.760	0.27	0.341	0.986	0.089	2.129	0.151	0.55
Frequency distractors	0.014	0.100	0.004	0.787	0.860	0.232	3.957	**0.001**	1.122

“/” = no Post-Hoc test specified

^a^Significant *p* values are shown in bold

**Table 8:** Post-Hoc Tukey’s test between clinical groups

Dependent variable	SCZ–ADHD			SCZ–ASD			ADHD–ASD		
	*t*_99_	*p*^a^	*d*	*t*_99_	*p*^a^	*d*	*t*_99_	*p*^a^	*d*
Mean % correct responses	3.555	**0.003**	0.721	3.686	**0.002**	0.746	0.206	0.997	0.175
Mean % missing data	2.216	0.126	0.563	2.542	0.06	0.604	0.393	0.979	0.1
Mean manual RT	2.635	**0.047**	0.67	4.547	**< 0.0001**	1.18	2.132	0.15	0.588
SD manual RT	1.171	0.646	0.339	3.656	**0.002**	1.046	2.733	**0.037**	0.727
Mean initiation of search	1.181	0.271	0.615	0.36	0.984	0.089	1.561	0.405	0.422
SD initiation of search	1.401	0.502	0.441	0.435	0.972	0.106	1.982	0.202	0.469
Frequency fixations	1.277	0.58	0.352	4.129	**< 0.0001**	1.152	3.137	**0.012**	0.803
Mean fixation duration	0.7	0.987	0.418	1.015	0.741	0.355	1.861	0.252	0.649
Mean of SD fixation duration	2.035	0.182	1.12	0.365	0.983	0.115	2.587	0.053	0.846
Mean search	2.019	0.188	0.53	4.384	**< 0.0001**	1.156	2.617	**0.049**	0.712
SD search	1.56	0.406	0.448	4.078	**0.001**	1.215	2.776	**0.033**	0.736
Mean scanning time	1.785	0.309	0.471	4.234	**< 0.0001**	1.151	2.754	**0.035**	0.734
SD scanning time	1.892	0.238	0.52	4.107	**< 0.0001**	1.214	2.452	0.074	0.686
Frequency distractors	0.726	0.886	0.203	3.607	**0.003**	0.963	3.15	**0.011**	0.888
Mean scanning pattern	0.86	0.825	0.235	3.717	**0.002**	1.07	3.135	**0.012**	0.89
SD scanning pattern	1.286	0.574	0.399	2.893	**0.024**	0.893	1.777	0.291	0.511
Mean post-search	3.294	**0.007**	0.838	2.382	0.087	0.55	0.94	0.783	0.262
SD post-search	2.516	0.064	0.711	0.999	0.75	0.249	1.613	0.376	0.466

^a^Significant *p* values are shown in bold

*ASD* paralleled superior performance in some search phases with inferior performance in others (Tables 6, 7, 8). First, they did not differ from TD or ADHD on manual mean RT while being faster than SCZ and, descriptively, the fastest group. Conversely, they were slower than TD in initiating search, not compared to SCZ or ADHD while being the second slowest group, after SCZ. Moreover, they were faster than TD, ADHD and SCZ in search and scanning time, slower than TD in post-search, not compared to SCZ and ADHD, showing the second longest post-search duration, following SCZ. Second, they did not differ from TD, but were less variable than ADHD and SCZ on manual RT. Conversely, they were more variable than TD, similarly to SCZ and ADHD in initiating search and post-search. Finally, they were less variable than TD, ADHD and SCZ during search, and less variable than TD and SCZ during scanning time. Third, ASD presented the smallest average frequency of fixations and of fixated distractors, the shortest and least variable scanning pattern compared to TD, ADHD and SCZ. However, ASD did show longer and more variable single fixation duration compared to TD, but not compared to ADHD and SCZ.

Within the SCZ group, correlation analyses between each dependent variable and CPE revealed that only the mean initiation of search significantly correlated with antipsychotics’ dose (*r*_*(*20)_ = 0.52, *p* = 0.02, *d* = 1.20, *η*^*2*^ = 0.27), while for any other dependent variable non-significant correlations with small to intermediate effect size (0.04 ≤ *r*_*(*20)_ ≤ 0.35, 0.08 ≤ *d* ≤ 0.74) were found.

**Fig. 3:**
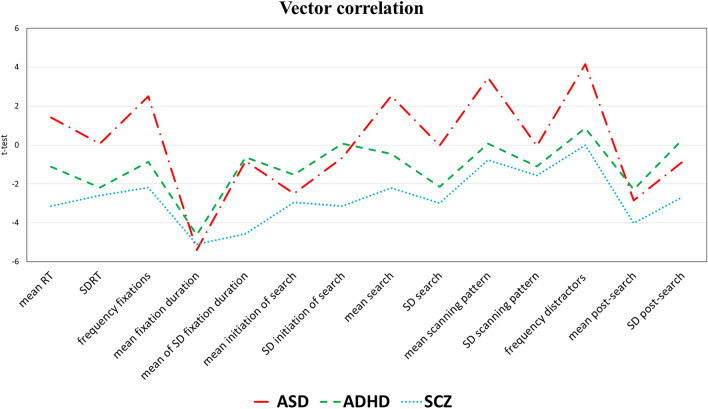
Vector correlations based on multiple comparisons of each clinical group versus TD. Vector correlation analyses included only dependent variables extrapolated from the correct trials. Mean and SD of scanning time were excluded, as the information they conveyed is included in search

The following results will broaden the scope to look at profiles of performance and how groups differ from or resemble each other in profile levels and the similarities of their shapes. Vector *levels* revealed that SCZ showed an overall greater impairment than ADHD (*t*_13_ = − 5.402, *p* < 0.0001, *d* = 1.15, *η*^*2*^ = 0.25*)* and, even more so, ASD (*t*_13_ = − 6.275, *p* < 0.0001, *d* = 1.33, *η*^*2*^ = 0.31), while ADHD were more impaired than ASD (*t*_13_ = − 2.426, *p* = 0.031, *d* = 0.57, *η*^*2*^ = 0.07). Vector *correlations* showed that the profiles of deviancy from controls for SCZ and ADHD (*r*_(14)_ = 0.683, *p* = 0.007, *d* = 1.87, *η*^2^ = 0.47), SCZ and ASD (*r*_(14)_ = 0.836, *p* < 0.0001, *d* = 3.04, *η*^2^ = 0.7) and ADHD and ASD (*r*_(14)_ = 0.745, *p* = 0.002, *d* = 2.23, *η*^2^ = 0.55) were highly positively correlated (Fig. 3).

## Discussion

In line with the idea of ASD, ADHD and schizophrenia as disorders with overlapping neurodevelopmental aetiologies and pathophysiologies, the present study investigated similarities and differences between these groups in a VS task challenging different cognitive functions, through the eye movement recordings, and resulting in process analyses of visual search. While the clinical groups differed in multiple ways across measures of processing speed, ISV and eye movement behaviour, as will be discussed below, similarities among clinical groups resulted in strong correlations of deviancy from controls, while the profiles of deviancy indicated SCZ as the most compromised group, followed by ADHD and then ASD.

### Processing speed

SCZ showed longer manual RT than both healthy controls and ADHD. The findings replicated the ones from the literature [10–12, 41] that had demonstrated less efficient serial search performance in patients with schizophrenia. Conversely, manual RT in ADHD was in the typical range. While the VS literature on children with ADHD often reported processing slowing during difficult serial search tasks [9, 10], conflicting results between the current studies and those from the literature might be due to age difference, and could point to performance normalisation with age in the ADHD group [42].

Conversely, ASD were faster than TD during search, scanning time and, descriptively, manual RT, in line with findings from the literature on manual RT [11]. By contrast, they showed delayed initiation of search and post-search, similarly to SCZ, suggesting different mechanisms involved in various search sub-phases.

Starting from initiation of search, according to the feature-integration theory [43], prior to the first saccade search-related processes might occur that require visual inspection of the search grid. It can be hypothesized that in patients with schizophrenia and ASD such time-consuming survey functions sub-optimally.

While the efficacy of an initial shift of attention is preserved when searching for highly salient targets, both in schizophrenia and ASD [44, 45], the present findings could propose that delay in initiating search is specific for conditions, where hardly distinguishable items are searched for. Such dissociation would suggest that is not the shift of attention per se but rather the top-down control of attention preceding the shift under more difficult task conditions that is impaired in the two groups. Similar conclusions come from studies on volitional as compared to visually-guided (or reflexive) saccades. While latency to initiate a visually-guided saccade is in the typical range in both clinical groups, latency to initiate a volitional saccade is slower in adults with schizophrenia and children with ASD, reflecting difficulties associated with activation of higher cognitive control processes [18, 46].

ADHD did not show any delay in initiating search. Despite the present results would suggest that the first stage of search is unimpaired in this clinical group, the only two other studies assessing the same construct produced conflicting results. While Karatekin and Asarnow [10] showed delayed search initiation in ADHD, Seernani et al. [47] did not. Despite Seernani et al. had provided similar findings, their search items were foreign language words, making the results difficult to compare with the current ones in light of the different perceptual properties of the stimuli as well as potential effect of language processing. Conversely, in Karatekin and Asarnow the search items (circle with intersecting vertical line) were more similar with the ones of the current study and conflicting results might, again express a moderating effect of age. Klein et al. [48] investigated age-related changes in saccade RT during the antisaccade task in 9–15-year-old ADHD and TD participants and found that the RT decrease in older as compared to younger participants was significantly higher in TD than ADHD, suggesting differential developmental processes in early adolescence in the two groups. Karatekin [49] replicated findings from Klein et al. in 12–18-year-old ADHD and TD participants and additionally reported that saccade RT of adolescents with ADHD did not differ from age-matched TD on the second task administration, pointing to ultimate normalisation of the ADHD performance in adolescence or early adulthood.

SCZ and ASD also shared longer post-search duration, suggesting common decision-making difficulties, which have already been documented separately for both groups [50, 51].

Duration of search was atypically long in SCZ. This pattern has been linked to difficulties in the top-down control of attention that is balancing attention towards relevant versus irrelevant items [52]. It could also result from visual information processing difficulties, in extracting complex features from the single items, because of visual perception deficits [12, 53] and/or difficulties in recalling information about the items from working memory [54, 55].

In contrast to schizophrenia, typical mean duration of search was found in ADHD, confirming the results from manual RT, initiation of search and percentage of correct responses. In parallel to a moderating effect of age, another potential explanation of these findings is that the level of task difficulty was suitable for subjects with ADHD to perform efficiently, in light of the relatively small number of distractors (15), target locations (eight out of 16) and number of targets (only one), while disruption in the VS performance in ADHD has been circumscribed to the easiest and most difficult task conditions [5].

Finally, search duration and scanning time were shorter than typical in ASD, expressing enhanced perceptual discrimination between target and distractors [56] or atypical attention as a tendency to “over-focus” [16].

### Characteristics of eye movements

SCZ, ADHD and ASD shared longer and more variable single fixation duration. Conversely, while SCZ and ADHD presented typical frequency of fixations, of fixated distractors and of total distance covered, ASD executed the smallest number of fixations, gazed less distractors, covered a smaller search area, compared to any other group. According to Elahipanah et al. [12] serial search relies on two processes: central discrimination, through which foveal information is used to discriminate the attended item from the search target and requiring focal attention, and peripheral selection, based on extra-foveal information used to select the next item to be foveated. Based on the measure of manual RT, the authors found intact visual guidance (peripheral selection) and impaired central discrimination deficit in schizophrenia. One could argue that single fixation duration is likely to express the efficiency of central discrimination, while the frequency of fixations and gazed distractors is an indicator of the efficiency of peripheral selection processes. The present results seem to suggest a deficit in central discrimination across the three groups, thus in extracting the features from an item, while peripheral selection is intact in SCZ, ADHD and ASD. Alternative explanations can be excluded based on current eye movement data. First, results do not suggest a working memory deficit, which would have implied frequent re-fixations. Second, results do not suggest enhanced perceptual discrimination, expressed by shorter single fixation duration. Third, results do not suggest abnormal shifting of attention, with consequent longer scanning time but reduced number of fixated distractors. To our knowledge, only one other study has measured eye movements during VS in schizophrenia [10], but did not include fixation-related measures. Conversely, comparison could be drawn based on studies on other experimental paradigms. Kurachi et al. [57] measured eye movements during the Picture Completion Test from the WAIS-R, where participants were asked to find the missing part in a picture, and found that patients with schizophrenia who failed in the task showed shorter scan path length and longer time for the first survey of the picture. Elahipanah et al. [12] administered the Symbol Digit Modalities Test, where participants needed to use a key area to find numbers corresponding to a series of presented symbols. Patients with schizophrenia spent longer time and fixated more frequently the key area in comparison to healthy controls. Finally, Sprenger et al. [58] presented patients with schizophrenia and TD with pictures of daily life situations and found longer but fewer fixations, shorter scan path lengths, fewer areas of interests fixated for longer times in schizophrenia than TD. Similarly, Karatekin and Asarnow [59] presented subjects with drawings from children’s books and found that patients with schizophrenia looked at fewer relevant regions, made longer fixations, tending to stare more at pictures when they were asked global questions. Overall, eye movement characteristics of the schizophrenia groups contributed to the “focal” in contrast to the “ambient” processing mode [60], which resembles the “local” in contrast to the “global” processing mode, that is a bias towards local or featural information rather than global properties of a stimulus [61, 62]. Finally, the overlap in findings across tasks could suggest its independence from task-specific aspects in schizophrenia.

Prolonged single fixation duration in ADHD suggests that extracting relevant characteristics from search items is more time consuming than in TD, while typical frequency of fixations and fixated distractors indicate preserved visual guidance processes. Noteworthy, and differently from SCZ, fixation duration paralleled typical duration of search and scanning time, suggesting that ADHD might have had more cognitive resources than SCZ to perform the task efficiently, while opting for a focal search strategy, like SCZ. Only two other studies have assessed VS in children with ADHD including fixation-related measures [48, 63]. Cui et al. [63] found prolonged first fixation duration but typical gaze duration (as the sum of all fixation durations), which resembles the finding of typical search duration found here, leading to atypical search processing limited to an early stage of search in ADHD. In contrast to the present finding, Seernani et al. [47] reported typical average fixation duration per trial in the ADHD group. Still, the non-irrelevant differences in the search item between the two studies—a foreign word in Seernani et al., a geometrically shaped item with gap in one out of four locations in the current study—may suggest that different search processes were prompted in the two studies, preventing a direct comparison of the findings. Preference for local processing in ADHD has been documented in one study. Song and Hakoda [64] presented participants with a classical and a computerised version of the Compound Digit Cancellation Test and found that subjects with ADHD attended more to local than global sub-tests and processed global information as they normally process local one. Conversely, Karatekin and Asarnow [59] and Ioannou et al. [27] found that patients with ADHD gazed static pictures similarly to TD, as shown by typical fixation duration across image’s areas of interest, which does not suggest a spontaneous local processing bias in ADHD. Further replication is required to explore if and under which conditions patients with ADHD prefer a local processing mode.

Among all groups, the shortest search and scanning time duration was found in ASD. While longer fixation duration point to suboptimal rather than enhanced central discrimination, the additional time on item in foveal vision, alongside adequate guiding attention towards the next item might have been determinant for the success in the task. Reduced frequency of fixations, fixated distractors and scanning pattern are in accordance with the observation that individuals with ASD can be disproportionately affected by contiguous items close to the current fixation, independently of their relevance (target versus distractors) [65]. It is possible that this might have contributed to prolonged fixation duration in the ASD group. Kaldy et al. [16] reviewed the VS literature on ASD and concluded that rather than enhanced perceptual low-level processes, it is atypical attention and specifically slowed attentional disengagement in the orienting system as measured by the classic ‘gap-overlap’ task that could explain enhanced visual search performance in ASD. Similarly, Colombo et al. [61] explained the local bias in healthy infants as atypical attention disengagement consequent to immature development of the orienting network. Based on present results, prolonged fixation duration in ASD might also be consequent to “sticky” visual attention, which causes them to disengage more slowly than healthy controls [66].

In contrast to the current finding, shorter fixation duration in ASD has been reported using the Embedded Figure Task (EFT), where participants are required to find a target inside a figure [62, 67, 68]. Jarrold et al. [67] found a strong correlation between the performance on the EFT and a feature type of search, that was opposite from controls, for whom the highest correlation was between the performance on the EFT and a serial type of search. While not explicitly stated by the authors, these results could suggest that patients with ASD may have employed a global processing mode when performing the EFT. Conversely, in the current study, the reduced dissimilarity between target and distractors might have required mainly local processing, which produced longer fixation durations in ASD (similarly to SCZ and ADHD). Recent work using various free viewing paradigms found that ASD exhibited reduced fixation duration on novel areas of the scene, tendency to look at fewer objects and consequent shorter scan path length, increased fixation duration on interesting objects and reduced fixations on uninteresting ones than TD, interpreted as attenuated global integrative processing style and enhanced detail-focused processing style when freely exploring a scene or when gazing high autism interest objects [23, 24, 69]. Taking these results altogether, local bias in ASD, advantageous on tasks that benefit from reduced distractibility, could explain suboptimal performance on tasks requiring shift of attentional engagement.

More generally, locally oriented processing style is manifest in multiple ways in ASD. It appears to be associated with symptoms of stereotyped and restricted behaviours, possibly mediated by the association of local processing and increased attention to details in daily life [70, 71] and with social interaction and communication deficits [71]. Despite being part of the constellation of autistic symptoms only, both restricted and repetitive behaviours [72, 73] and atypical social interactions [74, 75] have been variously related to the schizophrenia and the ADHD phenotypes.

### Intra-subject variability

Increased ISV in SCZ emerged from manual and eye movement measures and across search sub-phases. The strength of this finding confirms results from the literature, which shows increased ISV across fast decision tasks associated with various levels of cognitive demands [19, 76]. Overall, these studies established ISV as a measure of cognitive stability, consistent across cognitive tasks and different levels of task complexity, thus possibly reflecting a common underlying mechanism producing increased variability in different systems [77]. Unexpectedly, ADHD were significantly more variable in search initiation and duration of fixations only while being non-significantly more variable during remaining search phases, compared to TD. Given the robustness of the ISV findings in children with ADHD, participants’ age in the current study might suggest that the results express a moderating effect of age, coherent with evidence showing ISV progressive decrease from early childhood to adulthood [78]. Moreover, despite increased ISV in ADHD during VS has been linked to an early stage of search only [47], the current findings do not support such conclusion, in light of increased ISV of fixation duration. Conversely, the absence of post-search increased variability in ADHD is less surprising and could be explained by the reduced attentional requirements of deciding on the manual response, facilitated by the fact that the first button press indicated that the target item had been found and the second—which we have not included in the current analyses, where the target was located in the search grid [79].

Increased ISV in search initiation, post-search and single fixation duration was found in ASD, while they were the least variable group during search and scanning time. Despite ISV has been disproportionately less studied in ASD than ADHD and schizophrenia, literature that controlled for comorbidity between ADHD and ASD, comparing the performance of participants with ADHD only, ASD only and a comorbid group, concluded that the task-general increase in ISV reported for ADHD and comorbid groups does not extend to ASD [47, 80–82]. In light of ISV being increased in certain phases of search, and decreased in others, the current results seem to suggest that ISV in ASD, contrary to SCZ and ADHD, is specific to task and certain sub-phases of a task.

A possible explanation of differential ISV results in the three groups was proposed by Dinstein et al. [83], who hypothesized that ISV can be decomposed into distinct processes based on variability that relates to early versus late stages of task processing, variability that is specific to a local brain area versus shared across the entire brain and ultimately variability under present versus absent task conditions.

Increased ISV in adults with schizophrenia [84] and children with ADHD [85] has been related to resting state neural variability, possibly caused by abnormally unstable neuromodulation or to a dysfunction in adaptive gain [86], which could explain increased ISV in different processing stages and different systems of volitional control, suggesting a distributed neural network which modulates the adaptive regulation of performance common at SCZ and ADHD [87]. Conversely, while excessive neural variability was found when participants with ASD were presented with visual, auditory or tactile stimuli [83], it was not present in this clinical group when studying ongoing neural fluctuations [88], suggesting that increased ISV in ASD is associated with specific evoked processes but not with resting state fluctuations. Similarly, increased variability of P100 latencies expressing early visual response has been reported in ASD [89], increased variability of P300 latencies expressing later decision processing in ADHD [90], overall suggesting at least partially distinct forms of excessive variability in SCZ, ADHD and ASD in comparison to control individuals.

### Towards an NDD continuum?

Results from the process analyses enabled by eye movement recording during visual search highlight a pattern of both similarities and differences between SCZ, ADHD and ASD. While the slowing and trial-to-trial variability in SCZ is manifest across all sub-phases of the VS performance, it is specific for some in ADHD and is even reversed in ASD during search. Similarly, results on fixation-related measures highlight overlaps and dissociations in the way in which search items are processed.

At a cognitive level, deficit in attention sub-processes of focus and disengagement, as well as in decision processing, are central to the three disorders [91]. According to the current study, such deficits would be more pervasive in SCZ than the other two groups. Conversely, the sub-processes might function well in ASD under tasks conditions that require a detail driven strategy combined with a speeded performance, such as the case during VS.

At a pathophysiological level, similarities in cortical changes between SCZ, ADHD and ASD have been documented. Among the most relevant networks, the fronto-striatal system, responsible for decision-making processes requiring adaptive responding (initiation, execution, and withholding of responses), the fronto-parietal network, involved in top-down control, and the DMN, associated with task-irrelevant mental processes, have robustly been shown to be involved in ADHD, ASD and schizophrenia [92, 93]. The DMN has been particularly relevant in the understanding of abnormally high ISV across the three groups, because it interferes with neural circuits responsible for active task performance, while it is still unclear whether DMN activity levels are abnormal during rest [92, 94]. Furthermore, common structural abnormalities at the level of total brain volume, cortical thickness and structural brain connectivity have been found to be variously heritable, requiring further study of cognitive domains as potential trans-diagnostic endophenotypes [92, 95].

Similar profiles of impairment (or, in a few variables, strengths) between the clinical groups were also revealed by high vector correlations that corresponded to large effect sizes and were also found after removing trivial statistical dependencies between variables. If “impairment” is interpreted as a qualia that manifests in different cognition aspects, thus creating a profile of cognitive deviancy from “normality”, the substantial correlations (0.68 ≤ *r* ≤ 0.84) between the profiles of impairment in the clinical groups may suggest that they show the similar quality of cognitive impairment despite their different phenotypes.

In this continuum, SCZ showed the greatest level of impairment, ASD the smallest (0.57 ≤ *d* ≤ 1.33). If we consider a “continuum” as a quantitative grading of the same qualia, the present results suggest that patients with ASD, ADHD and SCZ take different positions on the same continuum. The coexistence of commonalities and differences between ASD, SCZ and ADHD suggests the multidimensionality of such continuum, re-imagined as a spectrum of an underlying loss of function (generalised deficit in SCZ, non-generalized deficits in ADHD and ASD) and gain of function (specific strengths in ASD, certain typical pattern in ADHD).

Vector correlations like the current ones depend upon various aspects on the specific tasks and measures included, implying that replication across different measures and tasks is a precondition for generalizability of the present findings. Moreover, although in the current study vector correlations were particularly high, future studies will have to explore also potential moderators of vector correlation profiles, such as psychometric reliability. Finally, the validity of the vector correlations in this context would be corroborated further if patterns of negligible or even negative correlations between different clinical groups could be found.

Some limitations should be noted. First, the size of the SCZ group was small, requiring replication. Second, SCZ were on current antipsychotics, whose effects cannot be excluded. Third, fatigue could have had an effect on the performance, given the long session duration.

## Conclusions

The present results found support for a pattern of similarities and differences between schizophrenia, ADHD and ASD. VS impairments seem to reflect a core generalised cognitive dysfunction in schizophrenia resulting from the three constructs of processing slowing, increased ISV and local processing bias. However, this finding was not entirely specific to schizophrenia, as ADHD and ASD also presented similar local bias and increased ISV in the initial stage of search, ASD in the post-search stage too. While increased focal attention in schizophrenia contributes to a suboptimal performance, it emerges as a successful strategy rather than a deficit in ADHD and is even advantageous for a speeded performance in ASD. Conversely, processing speed dissociates the three clinical groups being suboptimal in schizophrenia, enhanced in ASD, typical in ADHD. ISV has shown to be a powerful measure in dissociating reaction time of ADHD versus TD [20, 96] and schizophrenia versus TD [19, 77], across tasks, leading to hypothesize a similar reaction time distribution deviance in ADHD and schizophrenia. The present results only partially confirm such hypothesis within the study of VS, they suggest that schizophrenia presents the highest increase in ISV, but that the deficit in ADHD is limited to search initiation and fixation duration, while it extends to the other phases only descriptively. Results also support the hypothesis of increased ISV in ASD, in specific phases of search, while being reduced in others. An ASD dysfunction in pre-search and post-search and ADHD dysfunction in pre-search might suggest that ISV in ASD is related to planning and decisional processes, in ADHD to planning processes, leading to overlaps but also differences between ADHD and ASD, while dysfunction in schizophrenia is generalised. Furthermore, eye movement abnormalities point to a common attentional deficit, but present in different gradients, emphasizing the unique contribution of such methodology. Overall, findings support the shift towards dimensionality in recent nosological thinking, where the degree of neurodevelopmental impairment appears as a recognizable feature. Although preliminary, these results underscore the need for a simultaneous consideration of neurocognitive deficit profiles in potentially pathophysiologically related neurodevelopmental disorders, such as schizophrenia, ADHD and ASD.

## Data Availability

All authors are willing to share data and material to provide transparency.

## Code availability

All authors are willing to share codes to provide transparency.
